# A Remarkable Coincidence

**Published:** 1892-06

**Authors:** 


### A REMARKABLE COINCIDENCE.

Some time ago a lady in London wished to write to a friend in America whose address she did not know. The only means she had of procuring the address was to write to a mutual friend, who also lived in America. This she accordingly did, and the letter was duly dispatched. The ship which carried the letter was wrecked and the mails were for a time lost. They were eventually recovered and brought back to England, the letters, now much damaged by sea water, being returned through the dead letter office to the senders. The letter in question was sent back to the lady, who naturally examined it minutely.

To her surprise she found that another letter had become closely stuck to it. Holding up the twofold missive to the light, she deciphered the address on the one which was stuck to her own. It was a letter addressed to the friend to whom she had wished to write, and to discover whose whereabouts her own letter had been dispatched. Her letter thus literally brought back its own answer.—*Leeds* (*Eng.*) *Mercury.*

